# HDAC6: A Novel Histone Deacetylase Implicated in Pulmonary Arterial Hypertension

**DOI:** 10.1038/s41598-017-04874-4

**Published:** 2017-07-03

**Authors:** Olivier Boucherat, Sophie Chabot, Roxane Paulin, Isabelle Trinh, Alice Bourgeois, François Potus, Marie-Claude Lampron, Caroline Lambert, Sandra Breuils-Bonnet, Valérie Nadeau, Renée Paradis, Elena A. Goncharova, Steeve Provencher, Sébastien Bonnet

**Affiliations:** 1 Pulmonary Hypertension and Vascular Biology Research Group, Institut Universitaire de Cardiologie et de Pneumologie de Québec, Université Laval, Department of Medicine, Québec, Canada; 2 Pittsburgh Heart, Lung, Blood and Vascular Medicine Institute, University of Pittsburgh, Pittsburgh, PA USA

## Abstract

Pulmonary arterial hypertension (PAH) is a vascular remodeling disease with limited therapeutic options. Although exposed to stressful conditions, pulmonary artery (PA) smooth muscle cells (PASMCs) exhibit a “cancer-like” pro-proliferative and anti-apoptotic phenotype. HDAC6 is a cytoplasmic histone deacetylase regulating multiple pro-survival mechanisms and overexpressed in response to stress in cancer cells. Due to the similarities between cancer and PAH, we hypothesized that HDAC6 expression is increased in PAH-PASMCs to face stress allowing them to survive and proliferate, thus contributing to vascular remodeling in PAH. We found that HDAC6 is significantly up-regulated in lungs, distal PAs, and isolated PASMCs from PAH patients and animal models. Inhibition of HDAC6 reduced PAH-PASMC proliferation and resistance to apoptosis *in vitro* sparing control cells. Mechanistically, we demonstrated that HDAC6 maintains Ku70 in a hypoacetylated state, blocking the translocation of Bax to mitochondria and preventing apoptosis. *In vivo*, pharmacological inhibition of HDAC6 improved established PAH in two experimental models and can be safely given in combination with currently approved PAH therapies. Moreover, *Hdac6* deficient mice were partially protected against chronic hypoxia-induced pulmonary hypertension. Our study shows for the first time that HDAC6 is implicated in PAH development and represents a new promising target to improve PAH.

## Introduction

Pulmonary arterial hypertension (PAH) is a complex and multifactorial disease characterized by a progressive elevation of pulmonary vascular resistance, due to a cancer-like proliferative and apoptosis-resistant phenotype of pulmonary artery (PA) cells including smooth muscle cells (PASMCs) and endothelial cells (PAECs)^1^. This ultimately leads to right ventricular (RV) failure and premature death. Despite recent advances in molecular pathogenesis and treatment, none of the current treatment strategies cures this devastating condition^2^. Therefore, the identification and characterization of new targets specifically implicated in this pathological state and simultaneously disabling more than one mechanism of disease development and progression is a pressing need^3^. Interestingly, the documentation of numerous similarities shared by PAH and cancer cells^4–7^, brings an emerging paradigm in PAH pathology, opening to the possibility of exploiting therapeutic strategies used in cancer to treat PAH.

It is now established that PAH cells react to a hostile environment with adaptive and cytoprotective responses, allowing them to survive and proliferate and leading to intense remodeling of distal PAs. Central to these strategies are the over-activation of the DNA repair machinery^8–10^, the metabolic switch associated with resistance to mitochondrial-mediated cell death^11, 12^, the overexpression of molecular chaperones coping with the increasing number of misfolded proteins^13^, and the promotion of their clearance by autophagy^14^. Conversely, if stress stimuli go beyond a certain threshold, a variety of pro-apoptotic pathways culminating in cell death ensue, prevailing over the cytoprotective arms of the stress response. Thus, inhibiting these “over-efficient” stress coping mechanisms offers the opportunity to selectively induce stress overload and reverse the proliferative and anti-apoptotic phenotype in PAH cells.

Accumulated evidence pointed to histone deacetylase 6 (HDAC6) as an important druggable stress surveillance factor through its implication in multiple adaptive mechanisms aiming to prevent or cope with stress^15–17^. Contrary to nuclear HDACs implicated in epigenetic regulation of transcription, HDAC6 is a mainly localized cytoplasmic deacetylase involved in “non-histone” functions^18^. HDAC6 is overexpressed in many cancers and HDAC6 inhibitors display beneficial effects in various experimental models of cancer that shares several features with PAH^19, 20^. Importantly, HDAC6 does not deacetylate histones, and accordingly, the anticancer effects of the HDAC6-specific inhibitors are not associated with disrupted epigenetic control of gene transcription^21^. In directly influencing the acetylation status of several key cytosolic proteins^16–18, 22^, HDAC6 was reported to control numerous processes, impacting cell migration, proliferation and survival, all of which are important features of PAH^1, 23^. Indeed, HDAC6 promotes DNA repair and depletion or inhibition of HDAC6 sensitizes transformed cells to DNA damaging agents such as etoposide and doxorubicin^24–26^. Moreover, HDAC6 was reported to exert a protective role when cells are faced to stress in coordinating the clearance of misfolded protein aggregates prior to their engulfment in autophagosomes^27, 28^ and by preventing the translocation of apoptotic signaling proteins from the cytosol to the mitochondria^16, 25^. As a consequence, HDAC6 has emerged as a regulator of cell response to cytotoxic assaults. We therefore hypothesized that, as observed in many cancers, HDAC6 is overexpressed in PAH contributing to proliferation and resistance to apoptosis of PASMCs and that selective HDAC6 inhibition may represent a promising novel approach for the treatment of PAH.

## Results

### Increased expression of HDAC6 in human PAH and experimental models

**Figure 1:**
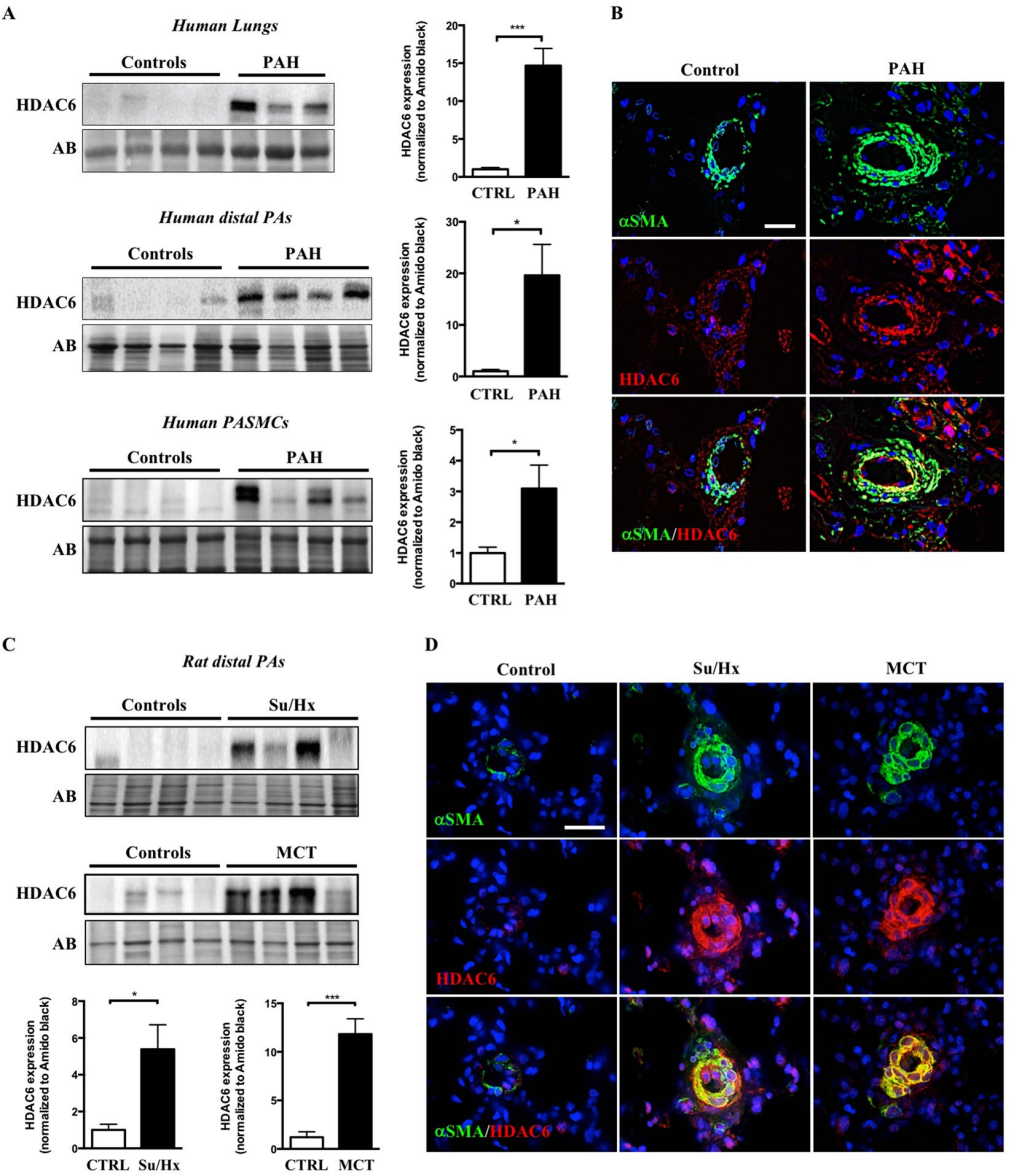
HDAC6 is overexpressed in lungs and distal pulmonary arteries (PAs) from PAH patients and animal models. (**A**) Representative Western blots and corresponding densitometric analyses of HDAC6 expression in lung tissues, distal PAs and isolated PA smooth muscle cells (PASMCs) from control (n = 4–8) and PAH patients (n = 4–12). (**B**) Double immunofluorescence staining for αSMA (green) and HDAC6 (red) and DAPI nuclear staining in lungs from control donors (n = 5) and PAH patients (n = 5), confirming the overexpression of HDAC6 in remodeled PAs. (**C**) Representative Western blots and corresponding densitometric analyses of HDAC6 expression in distal PAs of non-treated rats (n = 5 for each model) as well as rats exposed to Sugen-hypoxia (Su/Hx, n = 6) or monocrotaline (MCT, n = 5). (**D**) Double immunofluorescence staining for αSMA (green) and HDAC6 (red) and DAPI nuclear staining showing increased expression of HDAC6 in remodeled distal PAs after Su/Hx exposure or MCT injury compared to non-treated rats (n = 5 per group). Protein expression was normalized by Amido black (AB). Scale bar = 25 μm *P < 0.05 and ***P < 0.001.

Expression level of HDAC6 was measured by Western blot in lungs (n = 7–12) and distal PAs (n = 4–5) from control and PAH patients (Fig. 1A). As shown, HDAC6 was either not detected or barely detectable in lung tissues and distal PAs form control subjects, but was readily visualized in lung tissues from PAH patients. To confirm our results and identify the cell type responsible for augmented HDAC6 expression in human PAH lungs, we performed dual-immunofluorescence staining for alpha-smooth muscle actin (αSMA) and HDAC6. We noted that immunoreactivity for HDAC6 was considerably stronger in the lungs from PAH patients compared to the control lungs with a marked increase in PASMCs from the remodeled distal pulmonary arteries, as observed by the high degree of overlap between these two staining patterns (Fig. 1B). Similar findings were observed in the MCT and Su/Hx models (Fig. 1C and D). To further substantiate our findings, we analyzed the expression level of HDAC6 in primary cultured human PAH-PASMCs. In agreement with the above results, HDAC6 protein expression but not mRNA was robustly increased in PAH-PASMCs compared to control cells (Fig. 1A and Supplementary Figure S1). Interestingly, the acetylation level of α-Tubulin, a substrate of HDAC6^18^, was not changed between control and PAH-PASMCs (Supplementary Figure S1) suggesting that the increased activity of HDAC6 in these cells is counterbalanced by histone acetyltransferases as previously observed in other diseases^29^. In addition to PAH-PASMCs, HDAC6 was overexpressed in PAH-PAECs and PAH-RV as well as in the RV of rats exposed to Su/Hx and MCT (Supplementary Figure S1 and S2).

### Heat Shock Protein 90 (HSP90) regulates HDAC6 expression in PAH-PASMCs

**Figure 2:**
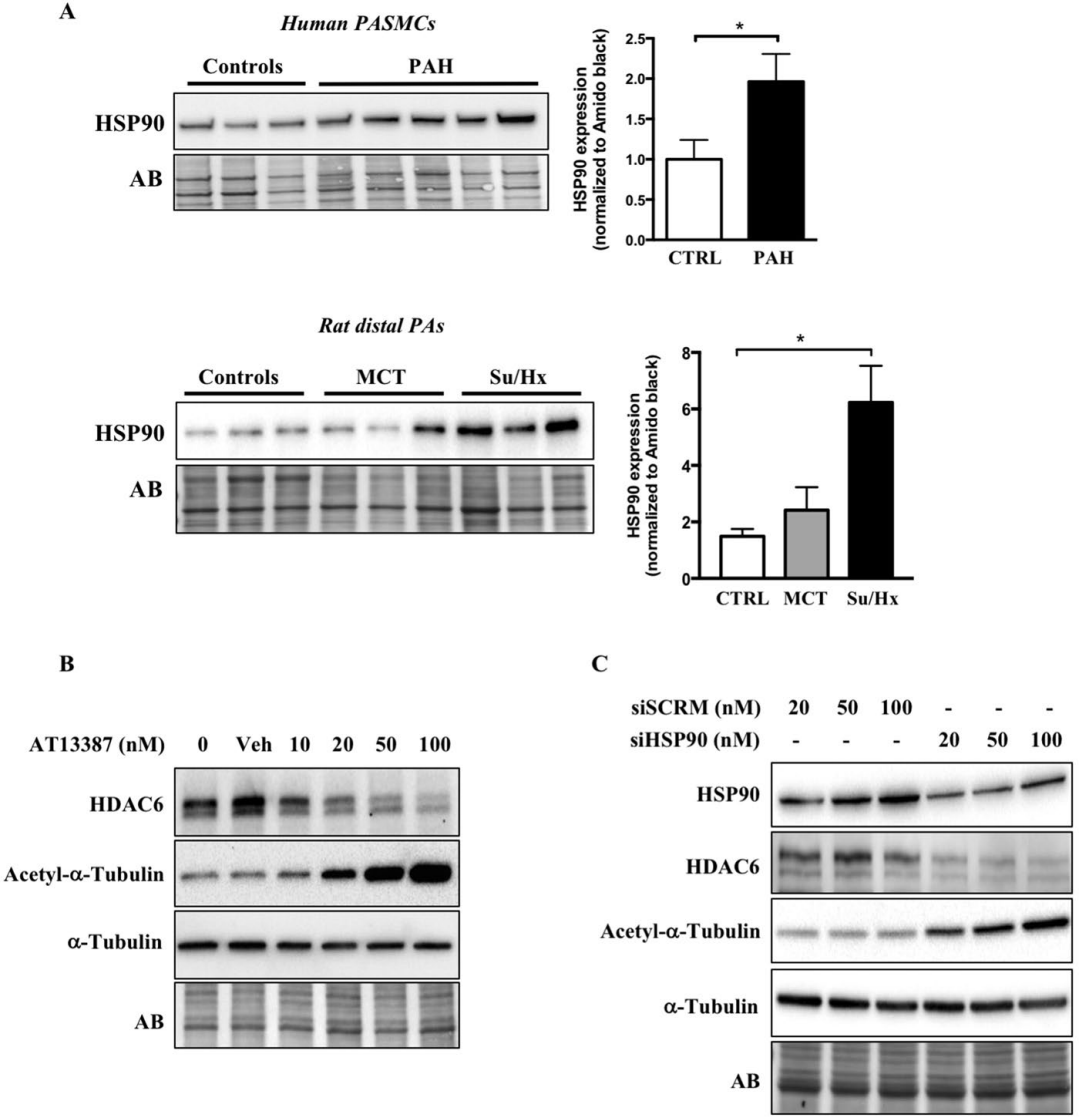
HSP90 is increased in PAH-PASMCs and stabilizes HDAC6 expression. (**A**) HSP90 expression was assessed by Western blot in pulmonary artery smooth muscle cells (PASMCs) from control (n = 3) and PAH patients (n = 5) and distal PAs from control (n = 3), MCT (n = 3) and Su/Hx (n = 3) rats. Protein band densitometry is reported in the corresponding graph. (**B**) Expression of HDAC6, acetylated-α-Tubulin, and α-Tubulin was assessed in PAH-PASMCs by Western blot after treatment or not with a pharmacological HSP90 inhibitor (AT13387, 10–100 nM for 48 hours) or vehicle (dimethyl sulfoxide). (**C**) Expression of HSP90, HDAC6, acetylated-α-Tubulin, and α-Tubulin was assessed in PAH-PASMCs by Western blot after treatments with a siHSP90 or siSCRM (20–100 nM for 48 hours). Blots are representative of two independent experiments. *P < 0.05.

To identify the mechanism responsible for HDAC6 overexpression in PAH-PASMCs, we focused our attention on HSP90. HSP90 is a central coordinator of an extensive array of cellular pathways upon stress through the stabilization of a wide range of proteins, which allows cells to survive and efficiently adapt to harmful conditions^30^. Given that HSP90 was recently found to be up-regulated in PAH^13^ and stabilizes HDAC6 in cancer cells^13, 22^, we thus hypothesized that the same mechanisms operate in PAH-PASMCs. We first measured HSP90 expression in isolated PASMCs from control and PAH patients as well as distal PAs from MCT and Su/Hx rat models. As observed for HDAC6, HSP90 was up-regulated in human and experimental PAH (Fig. 2A). To determine whether the up-regulation of HSP90 accounts for HDAC6 stabilization and thus its overexpression in PAH-PASMCs, pharmacological inhibition of HSP90 activity with AT13387 or 17-AAG or siRNA-mediated knockdown of HSP90 was performed. AT13387 (Fig. 2B) or 17-AAG (Supplementary Figure S3) treatments dose-dependently reduced HDAC6 protein abundance, correlating with a progressive acetylation of α-Tubulin. Similarly, HSP90 silencing resulted in a significant decrease in HDAC6 protein expression in PAH-PASMCs when compared with scrambled siRNA (Fig. 2C). Taken together, these findings support our hypothesis that increased expression of HSP90 in PAH-PASMCs maintains HDAC6 protein abundance and activity.

### HDAC6 inhibition recues the pro-proliferative, anti-apoptotic and pro-migratory phenotype of PAH-PASMCs

**Figure 3:**
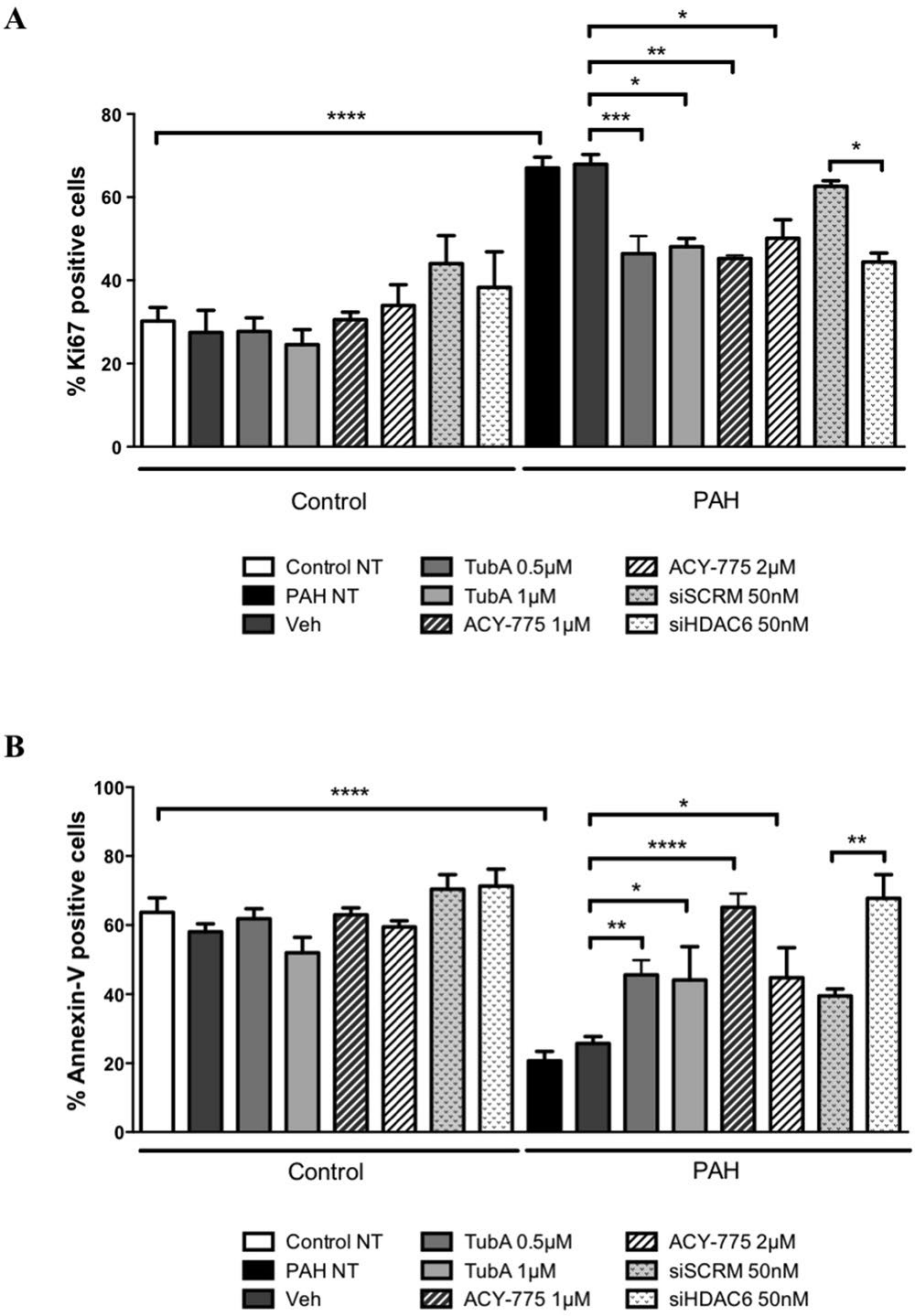
Inhibition of HDAC6 diminishes PAH-PASMC proliferation and resistance to apoptosis. (**A**) Proliferation (Ki67) was measured after treatments of PAH-PASMCs with two pharmacological HDAC6 inhibitors (Tubastatin A and ACY-775) or vehicle (DMSO), as well as siHDAC6 or siSCRM (50 nM) for 48 hours. (**B**) Apoptosis was similarly assessed following HDAC6 inhibition. *P < 0.05, **P < 0.01, ***P < 0.001 and ****P < 0.001. Experiments were performed in triplicate in 3 control and 4 PAH-PASMC cell lines.

The exaggerated migration, proliferation and survival of PASMCs is responsible for vascular remodeling in PAH^1^. Because HDAC6 has pro-migratory, pro-proliferative and anti-apoptotic functions in various cancer cell types, we investigated whether HDAC6 contributes to these functions in PAH-PASMCs. To this end, *in vitro* loss-of-function approaches using molecular and pharmacological tools were conducted. Inhibition of HDAC6 activity in PAH-PASMCs was first achieved using Tubastatin A (TubA) and ACY-775, two pharmacological inhibitors of HDAC6^31, 32^ as well as by small interfering RNA. PAH-PASMCs exposed to TubA or ACY-775 displayed a dose-dependent increase in the amount of acetylated α-Tubulin^18^ without affecting the total levels of α-Tubulin and the global degree of histone H3 acetylation, thus confirming the selectively of these inhibitors for HDAC6 (Supplementary Figure S4). Similar findings were observed following HDAC6 knockdown (Supplementary Figure S4). As expected, PAH-PASMCs had a significantly greater proliferation rate (as assessed by Ki67 labeling) and were more resistant to apoptosis (as assessed by Annexin V assay) when compared to control PASMCs. Inhibition of HDAC6 by TubA, ACY-775 or siHDAC6 significantly decreased PAH-PASMC proliferation and resistance to apoptosis (Fig. 3 and Supplementary Figure S4). Importantly, inhibition of HDAC6 had no effect on control PASMCs (Fig. 3), indirectly validating our above findings showing that HDAC6 is not or weakly expressed in these cells. Moreover, we found that pharmacological or molecular inhibition of HDAC6 reduced PAH-PASMC migration, as assessed by scratch wound assay *in vitro* (Supplementary Figure S5).

### HDAC6 inhibition causes Bax-induced cell death by increasing acetylation of cytosolic Ku70

**Figure 4:**
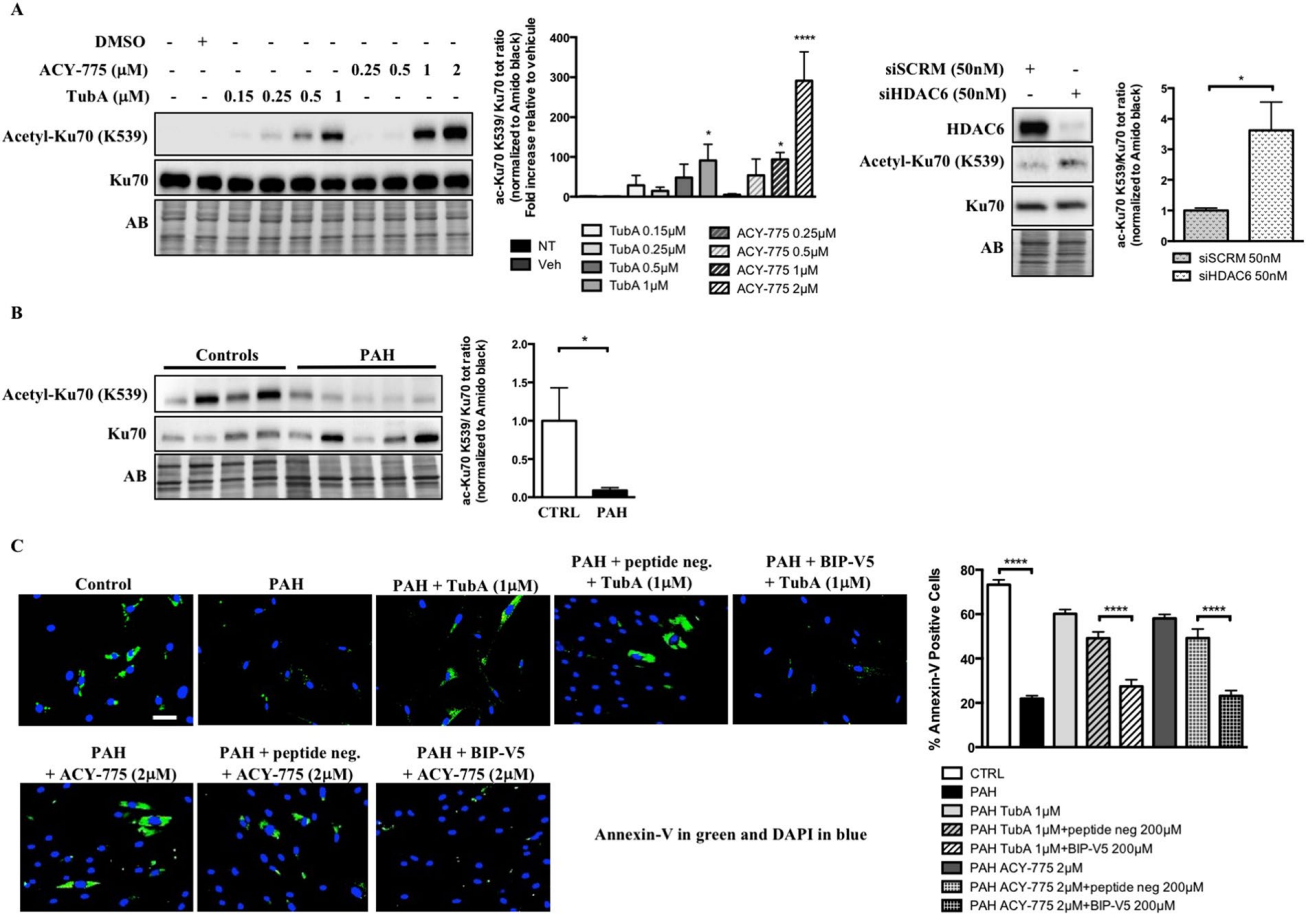
HDAC6 inhibition in PAH-PASMCs causes Bax-induced cell death by increasing acetylation of cytosolic Ku70. (**A**) Representative Western blots of acetyl(K539)-Ku70 and total Ku70 in PAH-PASMCs after treatments with two HDAC6 inhibitors Tubastatin A and ACY-775 or siHDAC6 for 48 hours. Data were expressed as the ratio of acetyl(K539)-Ku70/total Ku70. (**B**) Expression of acetylated(K539)-Ku70 and Ku70 was assessed in distal PAs from control (n = 4) and PAH patients (n = 5) by Western blot. Data were expressed as the ratio of acetyl(K539)-Ku70/total Ku70. (**C**) Apoptosis was measured using Annexin V staining (green) in control (n = 3 cell lines) and PAH-PASMCs (n = 3 cell lines) pre-treated or not with Bax inhibitory peptides (BIP-V5, 200 μM) or control peptides before being exposed to indicated HDAC6 inhibitors for 48 hours. Representative immunofluorescence images of apoptotic Annexin-V-positive cells (green) stained for DAPI (blue) and corresponding quantification are shown. Scale bar = 50 μm. Protein expression was normalized by Amido black (AB). ***P < 0.001 and ****P < 0.001.

We and others have previously demonstrated that mitochondria from PAH-PASMCs exhibit abnormalities rendering these cells less sensitive to apoptotic stimuli^4, 11^. Several cytosolic acetylated proteins have been identified as direct target of HDAC6. Among these, we paid particular attention to Ku70, a multifunctional protein for which acetylation correlates with susceptibility to mitochondrial-dependent apoptosis^16, 33, 34^. To demonstrate that HDAC6 inhibits Ku70 acetylation, enhancing Ku70-Bax binding and preventing stress-induced apoptosis, we first measured the acetylation level of Ku70 at lysine 539 (a critical acetylation site that influences Ku70-Bax binding)^16, 34^ in PAH-PASMCs exposed to HDAC6 inhibitors. As assessed by Western blots, TubA, ACY-775 and siHDAC6 treatments increased acetylation of Ku70 in a dose-dependent manner (Fig. 4A), suggesting Bax release and translocation to mitochondria. A similar finding was observed after inhibition of HSP90 confirming our previous results showing that HSP90 promotes HDAC6 expression (Supplementary Figure S6). Because HDAC6 efficiently deacetylates K539 *in vitro*, we thus hypothesized that acetylation level of Ku70 is abnormally low in PAH cells. To examine this hypothesis, we measured the level of acetylated(K539)-Ku70 levels in human PAs from control and PAH patients. We found that the acetylated(K539)/total Ku70 ratio was reduced in PA cells from PAH patients compared to their normal counterparts (Fig. 4B). To demonstrate that inhibition of HDAC6 is accompanied by translocation of Bax to the mitochondria, serum-starved PAH-PASMCs treated or not with TubA or ACY-775 for 48 hours were subjected to immunofluorescence labeling for Bax and mitochondria were stained with MitoTracker Red. We found that consistent with our previous finding showing that inhibition of HDAC6 is associated with decreased resistance to apoptosis, inhibition of HDAC6 in PAH-PASMCs resulted in a greater colocalization of Bax with MitoTracker (Supplementary Figure S7). To further strengthen the role of HDAC6 in the context of Bax-mediated cell death, PAH-PASMCs were pre-treated with a cell permeable Bax-inhibiting peptide (BIP)-V5 designed from the Bax inhibiting domain of Ku70^35^ followed by HDAC6 inhibition under starvation conditions. Pretreatment with BIP-V5 significantly rescued cells from apoptosis induced by HDAC6 inhibition, whereas no effect was detected with a negative control peptide (Fig. 4C). Taken together, these results indicate that HDAC6 protects PAH-PASMCs from stress-induced mitochondrial apoptosis through Ku70 deacetylation and sequestration of Bax in the cytosol.

### Pharmacological inhibition of HDAC6 improves pulmonary hypertension in the Sugen/Hypoxia rat model and provides a therapeutic effect comparable to the combination of standard PAH therapies

**Figure 5:**
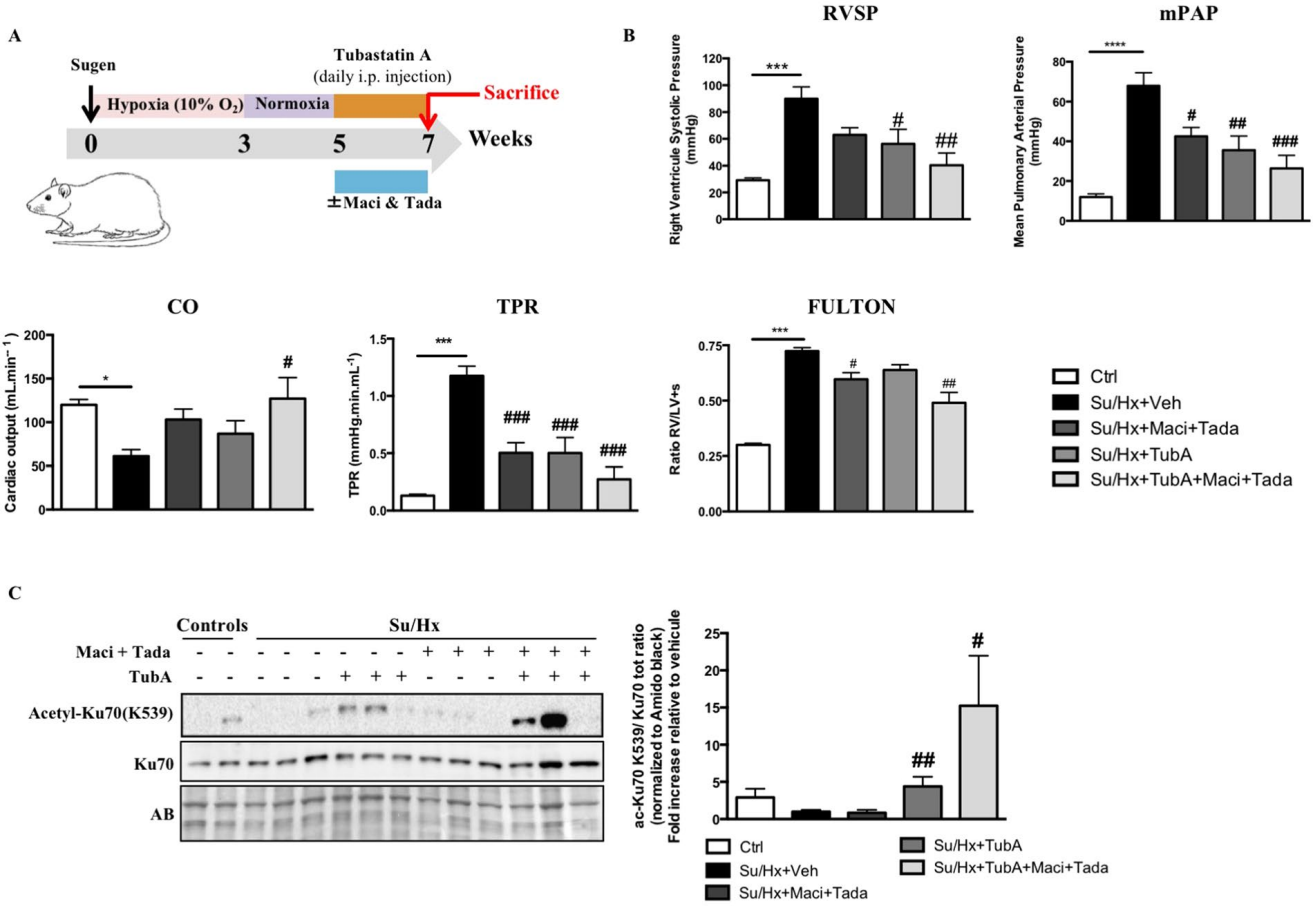
Tubastatin A (TubA) improves pulmonary arterial hypertension in the Sugen/Hypoxia (Su/Hx) rat model and provides a therapeutic effect comparable to the combination of standard PAH therapies. (**A**) Schematic of the experimental design. (**B**) RVSP, mPAP, CO, TPR and Fulton index were measured in control, Su/Hx + Veh (dimethyl sulfoxide), Su/Hx + Macitentan (30 mg/kg/d) + Tadalafil (10 mg/kg/d), Su/Hx + TubA (25 mg/kg/d) and Su/Hx + TubA + Macitentan + Tadalafil; n = 5 to 7 rats/group. (**C**) Expression of acetylated(K539)-Ku70 and Ku70 was assessed by Western blot in distal PAs from control and Su/Hx exposed rats treated or not with Tubastatin A (TubA, 25 mg/kg/d), combination of Macitentan (Maci, 30 mg/kg/day) and Tadalafil (Tada, 10 mg/kg/day) or combination of Tubastastatin A, Macitentan and Tadalafil for 2 weeks. TubA-treated Su/Hx rats display increased expression of acetylated(K539)-Ku70 confirming successful inhibitory effect of TubA on HDAC6 activity in distal PAs. Protein expression was normalized by Amido black and protein band densitometry was reported in the corresponding graph. *P < 0.05, ***P < 0.001 and ****P < 0.001 (*vs* control) and ^#^P < 0.05, ^##^P < 0.01, and ^###^P < 0.001 (*vs* Su/Hx + Veh).

**Figure 6:**
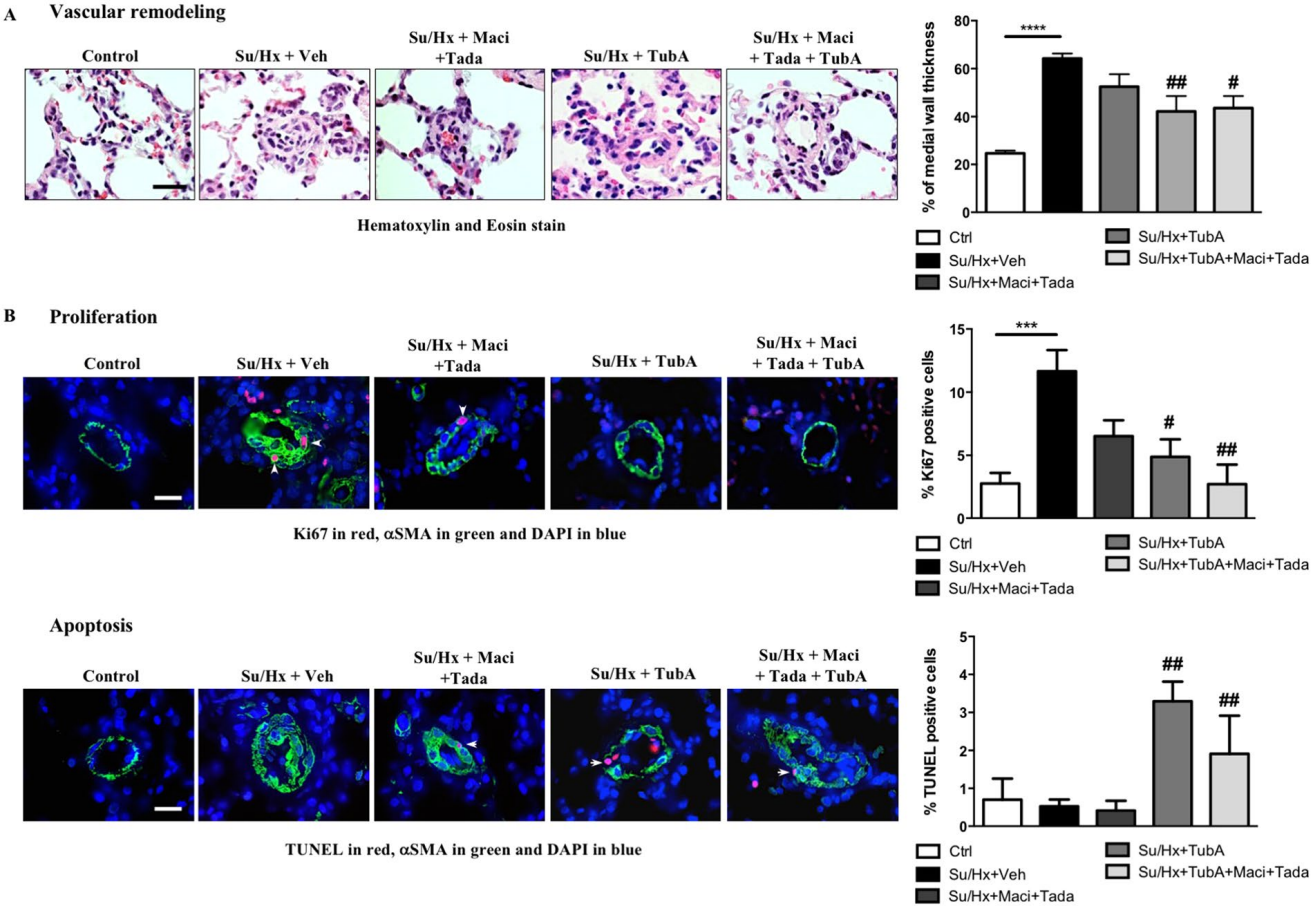
HDAC6 inhibition using Tubastatin A decreases vascular remodeling in Su/Hx-induced rats. (**A**) Representative images of distal pulmonary vessels and corresponding quantification of vascular remodeling as determined by the measure of the medial wall thickness using Hematoxylin and Eosin (H&E) stain and αSMA labeling. (**B**) Proliferation (Ki67) and apoptosis (TUNEL) were studied in lungs of control, Su/Hx-PAH + Veh, Su/Hx + Macitentan + Tadalafil, Su/Hx** + **TubA and Su/Hx + TubA + Macitentan + Tadalafil rats. Representative images of distal pulmonary vessels labeled with Ki67 (top) and TUNEL (bottom) in red. Vascular smooth muscle cells were labeled using alpha smooth muscle actin staining (green). Graphs represent the percentage of PASMCs positive for of Ki67- or TUNELin distal pulmonary vessels. Arrowheads mark positive cells. Scale bar = 20 μm; n = 5 to 7 rats/group (mean of 20 vessels/rat). **P < 0.01 (*vs* control) and ^#^P < 0.05, ^##^P < 0.01, and ^###^P < 0.001 (*vs* Su/Hx + Veh).

To validate our *in vitro* findings, we performed *in vivo* studies in the Sugen/Hypoxia (Su/Hx) rat model to test whether treatment with TubA is able to reverse PAH. Once PAH establishment was echocardiographically confirmed (five weeks post Sugen injection), TubA (25 mg/kg) was administered daily via intraperitoneal injection for two weeks (Fig. 5A). We demonstrated that TubA significantly improved RV systolic pressure (RVSP) and mean PA pressure (mPAP) in Su/Hx-treated rats, as assessed by right heart catheterization (Fig. 5B). Although not statistically significant, inhibition of HDAC6 tends to result in an increase in cardiac output (CO) and a decrease in right ventricular hypertrophy measured by Fulton index (Fig. 5B). Moreover, treatment with TubA resulted in a diminution of the total pulmonary vascular resistance (TPR) (Fig. 5B). To enhance the translational potential of TubA, we compared the efficacy of TubA alone or in combination with currently approved PAH therapies, namely Macitentan and Tadalafil. We found that TubA alone provides similar therapeutic effects than a combination of Macitentan and Tadalafil (Fig. 5B). Moreover, we confirmed that TubA administration decreased HDAC6 activity *in vivo* by assessing acetylated(K539)-Ku70 levels in dissected PAs (Fig. 5C). To determine whether the improvement of the hemodynamic parameters was due to reduced vascular remodeling of distal pulmonary vessels, vascular wall thickness was quantified by hematoxylin and eosin staining and αSMA labeling. As expected, Su/Hx induced a severe vascular remodeling as evidenced by vascular wall thickening comprising majority of αSMA positive cells. Medial hypertrophy of distal pulmonary vessels was greatly reduced following HDAC6 inhibition (Fig. 6). Consistently, the percentage of occluded pulmonary vessels was significantly lower in Su/Hx-treated rats given TubA than in those given vehicle (Supplementary Figure S8). We next validated our findings by measuring proliferation and apoptosis of distal PASMCs detected by αSMA labeling. In Su/Hx-challenged rats, proliferation of PASMCs was enhanced whereas virtually no apoptosis was detected as compared with controls. As observed *in vitro*, TubA suppressed proliferation and induced apoptosis in PASMCs (Fig. 6).

### Pharmacological inhibition of HDAC6 reverses pulmonary arterial hypertension in MCT rats

To further demonstrate the therapeutic benefit of TubA, we investigated whether the corrective effects observed in the Su/Hx model can be reproduced in a second model of PAH. As observed in the Su/Hx model, inhibition of HDAC6 with TubA significantly improved RVSP, mPAP, CO, TPR, RV hypertrophy, vascular remodeling (as assessed by medial wall thickness and percentage of occluded vessels) and cell proliferation/apoptosis imbalance in the MCT rat model (Supplementary Figure S9) reinforcing the view that HDAC6 is an essential component of vascular remodeling in PAH. Collectively, these results strongly support our hypothesis that HDAC6 promotes vascular remodeling in PAH and that its inhibition represents a new and promising avenue to tackle PAH.

### *Hdac6* loss of function in mice confers protection against chronic hypoxia-induced pulmonary hypertension

**Figure 7:**
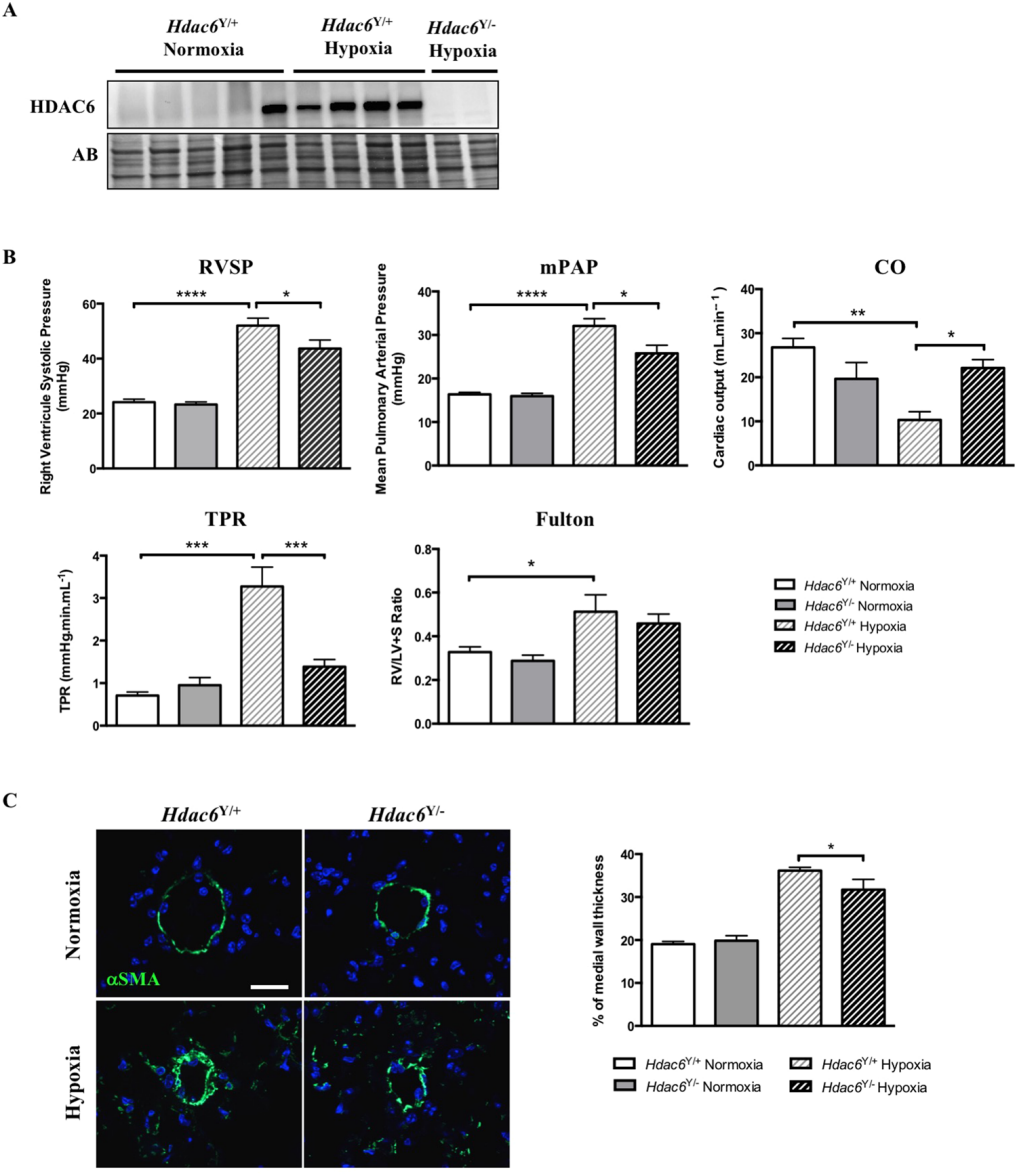
*Hdac6* loss of function in mice confers protection against chronic hypoxia-induced pulmonary hypertension. (**A**) HDAC6 expression was measured by Western blot in lungs from wild-type (*Hdac6* ^Y/+^) and *Hdac6* mutant (*Hdac6* ^Y/−^) mice exposed or not to hypoxia for 3 weeks. (**B**) RVSP, mPAP, CO, TPR and Fulton index were measured in wild-type and *Hdac6* mutant mice exposed to normoxia or hypoxia (10% O_2_) for 3 weeks. (**C**) Representative images of distal pulmonary vessels and corresponding quantification of vascular remodeling as determined by the measure of the medial wall thickness using αSMA labeling. (mean of 10 vessels/mice). Scale bar = 20 μm. Protein expression was normalized by Amido black (AB). n = 5 to 10 mice/group. *P < 0.05, **P < 0.01, ***P < 0.001 and ****P < 0.0001.

To test whether HDAC6 constitutes a necessary factor for pulmonary hypertension (PH) development, wild-type (*Hdac6* ^Y/+^) and *Hdac6* knockout mice (*Hdac6* ^Y/−^) were exposed to either normoxia or hypoxia (10% O_2_) for 3 weeks. We first validated the absence of HDAC6 in mutant mice and demonstrated that hypoxia exposure significantly increased HDAC6 expression in lungs from *Hdac6* ^Y/+^ mice (Fig. 7A). Contrary to our findings in human PAH and rat models, levels of HSP90 in whole lung homogenates were not increased by hypoxia (data not shown). As expected, loss of *Hdac6* function in normoxic and chronic hypoxic mice was accompanied by the hyperacetylation of α-Tubulin and Ku70 (Supplementary Figure S10). From a hemodynamic point of view, chronic hypoxia induced PH in both *Hdac6* ^Y/+^ and *Hdac6* ^Y/−^ mice (increased RVSP, mPAP, TPR and decreased CO) nonetheless; these effects were significantly attenuated in mutant mice (Fig. 7B). Note that no differences in all the hemodynamic measurements were seen between *Hdac6* ^Y/−^ and *Hdac6* ^Y/+^ mice (Fig. 7B). A trend to a decrease in RV hypertrophy measured by Fulton index was also seen in mutant mice (Fig. 7B). Finally, *Hdac6* ^Y/−^ mice had reduced pulmonary vascular remodeling, as assessed by αSMA labeling (Fig. 7C).

## Discussion

**Figure 8:**
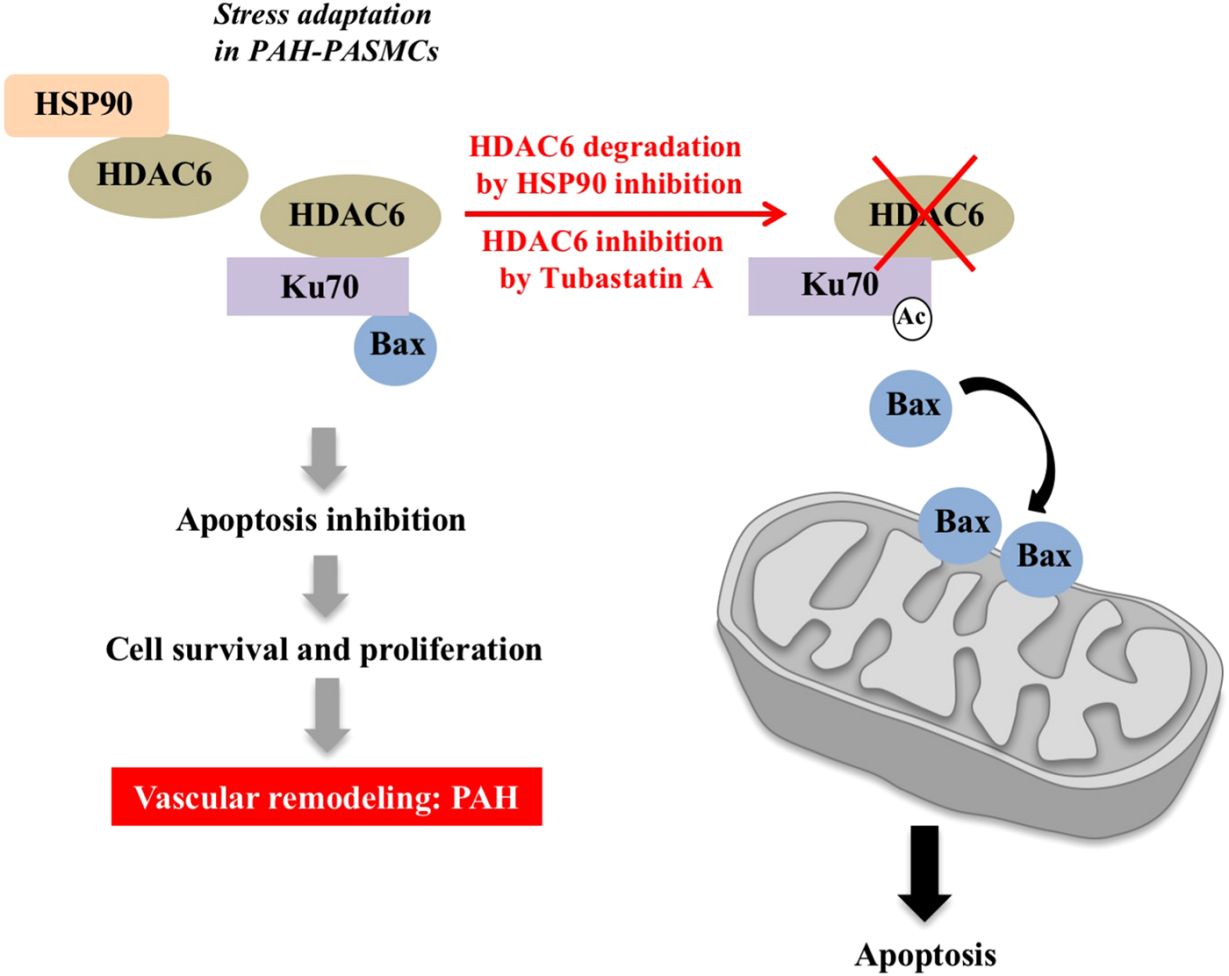
Proposed model depicting the molecular mechanisms by which HDAC6 promotes vascular remodeling in PAH. To deal with stressful conditions, HSP90 is upregulated in PAH-PASMCs and stabilizes HDAC6. HDAC6 maintains cytosolic Ku70 in a hypoacetylated state preventing Bax translocation to mitochondria and subsequent apoptosis. This results in increased cell survival contributing to vascular remodeling and PAH progression. Conversely, HSP90 or HDAC6 inhibition increases Ku70 acetylation and Bax release promoting mitochondrial membrane depolarization and programmed cell death.

In the present study, we found that HDAC6 is strongly up-regulated in lungs, distal PAs and isolated PASMCs from PAH patients and animal models. In agreement with previous findings pinpointing HDAC6 as a regulator of the cell protective responses^15^, we demonstrated that HDAC6 antagonizes apoptosis in PAH-PASMCs by maintaining Ku70 in a hypoactelyated state. Hypoacetylated Ku70 interacts with Bax and suppresses the apoptotic translocation of Bax to mitochondria, as shown in Fig. 8. More importantly, we demonstrated that its selective inhibition improved the pro-migratory, pro-proliferative and apoptosis-resistant phenotype of PAH-PASMCs *in vitro* sparing control cells and significantly improved established PAH in two relevant rat models.

Although the overall status of histone modification is largely unknown in PAH, nuclear or pan-HDAC inhibitors have also been exploited in PAH for their beneficial effects under several pathophysiological conditions like cancer and inflammation^36^. These investigations have yielded mixed results. Indeed, studies conducted in rat models of PH have reported positive impact of class I HDAC inhibitor on pulmonary vascular remodeling and RV hypertrophy^37, 38^. These results contrast with those documenting no therapeutic effects of broad-spectrum HDAC inhibitors in the Su/Hx-induced PAH rat model or even a deterioration of RV function in a rat model of RV hypertrophy^39, 40^. The unresolved controversies highlighted by these studies may reflect, at least in part, the unforeseeable side effects caused by nuclear HDAC inhibitors. Unlike most classical HDACs, which are located in the nucleus and deacetylate nuclear histones, HDAC6 is predominantly localized within the cytoplasm where it associates with non-histone substrates^18^. Therefore, our findings demonstrating therapeutic benefit of Tubastatin A in PAH models combined to the fact that HDAC6 has limited and nonlethal implication in physiological processes^41^ and is minimally expressed in lung control cells make it a promising candidate towards therapeutic implementation in human PAH.

Similarly to the lungs, HDAC6 is up-regulated in the RV of both PAH patients and experimental rats models of PAH (Su/Hx and MCT). Although its role in RV failure was not explored in the present study, previous studies in heart failure revealed that HDAC6 promotes cardiac fibrosis^42^ and its inhibition was associated with cardioprotective effects in a left ventricular pressure overload model^43^. This could thus explain the cardioprotective effects (better CO) of Tubastatin A in our models. Nonetheless, further studies are needed to fully elucidate the role of HDAC6 in RV failure.

Consistent with a recent report^13^, we demonstrated that HSP90 expression is increased in human PAH-PASMCs as well as in two rat models of PAH leading to HDAC6 stabilization. Interestingly, analysis revealed that hypoxia exposure in mice was not accompanied, by significant change in HSP90 expression in whole lung homogenates, while HDAC6 being up-regulated. This finding indicate that hypoxia alone is not sufficient to up-regulate HSP90 and suggests that HDAC6 regulation by HSP90 relies more on its activity level rather than its expression level. Although HDAC6 up-regulation is a common denominator of human PAH and rat models, the differences in HSP90 expression among the PAH models underscore the need of exploring different models in the same study because none of the animal models fully recapitulate all aspects of the disease^3^.

Our observations showing that inhibition of HDAC6 increases acetylation of Ku70 at lysine residue 539, known to abolish the ability of Ku70 to suppress Bax-mediated apoptosis^34^ and that Tubastatin A treatment enhances PAH-PASMC apoptosis, which is blocked by a Bax-inhibiting peptides derived from the Bax-binding domain of Ku70, strongly support the implication of Ku70 in vascular remodeling. These findings are in line with a previous study reporting that HDAC6 expression in neurobastoma cells maintains cytosolic Ku70 in a hypoacetylated state leading to Bax sequestration from the mitochondria and apoptosis evasion^16^. Interestingly, similar findings (increased HDAC6 and enhanced Ku70 acetylation in response to Tubastatin A) were found in PAH-PAECs suggesting the implication of HDAC6 in PAH-PAEC hyper-proliferation.

In addition to its cytoprotective functions in the cytosol, Ku70 is also involved in DNA double-stranded break and its acetylation level inversely correlates with its DNA repair efficiency^44^. Interestingly, *Ku70* deficient mice exhibit lung abnormalities characterized by emphysema and blood vessel occlusions^45^, two conditions intimately connected to defective DNA repair mechanisms and cell apoptosis. It is now well accepted that in the early stages of PAH, enhanced inflammation, oxidative stress and down-regulation of bone morphogenetic protein receptor type 2 (BMPR2) signaling cause irreversible accumulation of DNA damage and subsequent PAEC apoptosis^46^. During disease progression, sustained activation of the DNA repair machinery was identified as an adaptive response used by PAH cells to face stress, allowing them to survive and hyper-proliferate^8, 9^. Thus, it can be assumed that endothelial cell overgrowth and development of plexiform-like lesions in *Ku70* mutant mice is the consequence of initial apoptosis due to accumulation of DNA damage and subsequent selection of apoptosis-resistant proliferating cells. Although not explored in the present study, inhibition of HDAC6 in established PAH may impair nuclear Ku70-mediated DNA repair function, contributing to decreased PAH-PASMC DNA repair capacity and thus diminished cell proliferation and resistance to apoptosis.

Our results identified cytosolic Ku70 as a target of HDAC6 promoting PAH-PASMC survival. However, several other mechanisms mediated by HDAC6 could explain the abnormal pro-proliferative and anti-apoptotic phenotype of these cells. For instance, HDAC6 was reported to promote autophagy^27^ that is induced in human and experimental PAH^14, 47^. Although debated, autophagy is considered as a pro-survival mechanism against cellular stress, allowing removal of stress-induced damaged organelles and toxic protein aggregates^48^. In addition, HDAC6 interacts and deacetylates various other proteins including Survivin and HSP90 to carry out cancerous functions^49, 50^. Indeed, HDAC6 was reported to induce Survivin deacetylation promoting its cytoplasmic localization and thus its anti-apoptotic functions^50^. Given that we previously demonstrated that Survivin is overexpressed in PAH-PASMCs contributing to the development of PAH^7, 51^, HDAC6 inhibition may also contribute to improve PAH by reducing the cytoplasmic pool of Survivin leading to increased sensitivity to apoptosis. Moreover, inhibition of HDAC6 was reported to increase acetylation of HSP90 and thus weaken its chaperone-dependent functions^41, 49^. Interestingly, HSP90 stabilizes various proteins associated with malignant growth and proliferation such as proviral integration site for Moloney murine leukemia virus-1 (PIM-1), signal transducer and activator of transcription 3 (STAT3) and hypoxia-inducible factor 1 alpha (HIF-1α)^52, 53^, also found to be increased and implicated in PAH^6, 7, 11^. Based on these findings, it can be assumed that HDAC6 overexpression in PAH-PASMCs favors HSP90 activity contributing to the proliferative and anti-apoptotic phenotype of PAH-PASMCs. In agreement with this, inhibition of HSP90 was recently reported to improve pulmonary vascular remodeling in experimental PAH^13^. Nevertheless, as reported in cancer cells^54^, we found that Tubastatin A does not induce hyperacetylation of HSP90 in PAH-PASMCs (data not shown) indicating that the beneficial effects of HDAC6 inhibition seen in our models cannot be ascribed to a weakening of HSP90 function.

In conclusion, our results identify HDAC6 as a druggable regulator of PAH-PASMC resistance to apoptosis and offer novel insights into the molecular mechanisms governing vascular remodeling. Importantly, HDAC6 has limited and nonlethal implication in physiological processes^41^ and phase 1/2 clinical trials investigating HDAC6 inhibitors in cancer are currently ongoing (clinical trial NCT 01323751). Based on these findings, HDAC6 inhibition represents a new and promising avenue to treat PAH, avoiding the unforeseeable undesirable side effects caused by non-specific nuclear HDAC inhibitors.

## Methods

All experiments were performed with the approval of Laval University and the IUCPQ Biosafety and Ethics Committees, as well as in accordance with recent recommendations on optimal preclinical studies in PAH^3^.

### Human tissue samples

Experimental procedures using human tissues or cells conformed to the principles outlined in the Declaration of Helsinki. Tissues were obtained from patients who had previously given signed informed consent. Healthy lung tissues (controls) were obtained during lung resection for tumors. Lung samples were taken at distance from the tumor and demonstrated normal lung parenchyma. All the PAH tissues were from lung explants from transplant or early (“warm”) autopsy. PAH diagnosis was previously confirmed by right heart catheterization. PAH and control tissues were obtained from Respiratory Health Network tissue bank. PAH and control donor were matched for age and sex (Supplementary Table 1)^3^. Keep in mind that although usage of human tissues is an invaluable resource, they are commonly obtained from patients with end-stage PAH only and non-PAH samples are rarely obtained from true healthy donors.

### Reagents and inhibitors

ACY-775 (2-((1-(3-fluorophenyl)cyclohexyl)amino)-*N*-hydroxypyrimidine-5-carboxamide) was obtained from Acetylon Pharmaceuticals. Tubastatin A (TubA) and 17-AAG (Tanespimycin) were purchased from SelleckChem. AT13387 (Onalespib) was purchased from AdooQ Bioscience. In some experiments, 200 μM of Bax inhibitor peptide V5 (BIP-V5, MedChemExpress) or its corresponding control peptide (Millipore) was applied 16 hours before treatment with Tubastatin A or ACY-775.

### Cell culture and treatments

PAH-PASMCs (n = 5 cell lines) were isolated from small PAs (<1000 μm diameter) from PAH patients. Controls PASMCs (n = 4 cells lines) were either purchased from Cell Application or isolated from non-PAH patients as previously described^5, 8^. Cells were used at passages 4 to 9 for experiments. No changes in cell morphology were noted and PASMC phenotype was confirmed by α-smooth muscle actin staining, as previously done^5, 8^. PASMCs were grown in high-glucose DMEM supplemented with 10% Fetal Bovine Serum (Thermo Fisher Scientific) and 1% antibiotic/antimytotic (Thermo Fisher Scientific). TubA and ACY-775 were dissolved first in dimethyl sulfoxide (DMSO, Sigma-Aldrich) and then added to the culture medium (0.15–2 μM) immediately before use. siRNAs were transfected at a final concentration of 20–100 nM with Lipofectamine RNAiMAX (Thermo Fisher Scientific).

### *In vitro* proliferation and apoptosis measurements

PASMCs were cultured for 48 hours in 10% fetal bovine serum (FBS, a condition that is known to promote proliferation) or 0.1% FBS (a starvation condition that promotes apoptosis) in presence or absence of TubA, ACY-775, siHDAC6 or their respective controls. After treatment, cells were fixed with 4% paraformaldehyde in PBS at room temperature for 20 min. PASMC proliferation was measured by Ki67 labeling and apoptosis by AnnexinV assay, as previously performed^5, 6, 8, 55^. The total number of positive cells was calculated and divided by the total counter number of nuclei (DAPI), thus permitting the determination of percent values for each condition. At least 100 cells by experiment in 3 experiments were counted. All experiments were at least performed in triplicate.

### Cell migration assay

PAH-PASMCs were grown to confluence. The cells were exposed or not to TubA, ACY-775, siHDAC6 or their corresponding controls for 24 hours before a linear scratch was made using a sterile 1000-μL pipette tip. Detached cells were removed by repeated washes and fresh medium containing or not HDAC6 inhibitors was added. Wound closure was monitored during 24 hours by phase microscopy capturing images. Initial and remaining wound areas were determined with the aid of the image processing software «Image J» for calculation of the percentage of wound closure.

### Quantitative RT-PCR and immunoblotting

Total RNAs were extracted from control and PAH-PASMCs using TRIzol reagent (Invitrogen), as previously described^56^. Real-time RT-PCR was performed using a TaqMan RNA-to-CT 1-Step kit (Applied Biosystems) and a QuantStudio 7 Flex system (Applied Biosystems). HDAC6 expression was analyzed using the Taqman Gene Expression Assay (Assay ID Hs00997427_m1) from Applied Biosystems following the manufacturer directions. Reactions were run in triplicate and relative quantification was achieved with the comparative 2^−ΔΔ*C*t^ method by normalization with 18 s ribosomal RNA.

For Western blotting, protein lysates were prepared from healthy and PAH tissues or PASMCs in a 2% Chaps protein extraction buffer containing protease Inhibitor (Roche) and phosphatase Inhibitor Cocktails (Sigma). The protein concentration of the extracts was determined using Bradford reagent (Bio-Rad). Equal amounts of protein were separated by SDS gel electrophoresis, transferred to PVDF membranes and incubated with either 5% non-fat dry milk or 10% goat serum in TBS-T buffer, then membranes were incubated overnight at 4 °C with primary antibodies: HDAC6 (from Santa Cruz (1:100) for human samples and from Cell Signaling (1:1000) for rodent samples), Acetylated-α-Tubulin (Cell Signaling, 1:1000), α-Tubulin (Sigma, 1:5000), HSP90 (StressMarq, 1:2000), acetyl(K539)-Ku70 (St John’s Laboratory, 1:1000), Ku70 (Santa Cruz, 1:1000), acetyl-Histone H3 (Cell Signaling, 1:1000) in 3% BSA. Next, membranes were incubated with appropriate horseradish peroxidase (HRP)-conjugated secondary antibody (Promega, 1:10000) for 2 hours at room temperature in 5% non-fat milk or in 3% BSA. Antibodies were revealed using ECL reagents (Perkin–Elmer) and using the imaging Chemidoc MP system (Bio-Rad Laboratories). Protein expression was quantified using the Image lab software (Bio-Rad Laboratories) and normalized to Amido black (AB), as previously described^5, 8, 56^.

### Immunohistochemistry and immunofluorescence studies

For *in vitro* localization of Bax, PAH-PASMCs serum-starved in 0.1% FBS and exposed or not to HDAC6 inhibitors for 48 h were stained with the cell-permeable mitochondrion-selective dye MitoTracker Red (500 nM, Life Technologies), fixed, permeabilized, and stained with antibody specific for Bax (Abcam, 1:150) Images were acquired using a Carl Zeiss MicroImaging microscope. Colocalization between Bax and MitoTracker was estimated by the software using an algorithm that calculates the Pearson’s correlation coefficient.

Paraffin-embedded lungs were serially sectioned at 5 µm. Following citrate-based antigen retrieval, the sections were blocked with goat serum (5%) for 1 hour. Then, sections were incubated with primary antibodies at 4 °C overnight. Rabbit polyclonal anti-HDAC6 (1:400, Abcam), anti-Ki67 (1:400, Millipore), and mouse monoclonal anti alpha smooth muscle actin (αSMA, 1:200, Sigma) were used as primary antibodies. Proteins were detected using appropriate fluorescent-dye conjugated secondary antibodies (Thermo Fisher Scientific). Apoptotis was detected by *in situ* direct DNA fragmentation (TUNEL) assay, according to manufacturer’s instructions. The Cyanine 3 Tyramide Signal Amplification Kit (PerkinElmer) was used for HDAC6 detection. Images were obtained with a Zeiss ApoTome microscope. The percentage of Ki67 or TUNEL-positive PASMCs was calculated by examining 20 randomly selected distal pulmonary vessels (<50 μm) from each of 5 to 11 rats from each experimental group, as previously described^5, 8, 55^.

### Animal models

All animal protocols were approved by the Laval University and the IUCPQ Biosafety and Ethics Committes. The Sugen-hypoxia (Su/Hx) and the monocrotaline (MCT) PAH rat models were used in the present study. Male 250–350 g Sprague-Dawley rats (Charles River Laboratories) were used for both animal models. For the MCT model, rats were injected subcutaneously with 60 mg/kg of monocrotaline (Sigma). For the Su/Hx model, rats were injected with 20 mg/kg of SU5416 (Sigma) and put in hypoxia (10% O_2_) for 3 weeks. Once PAH was established (after 14 days for MCT and week 5 for Sugen and confirmed by echography), TubA (a specific HDAC6 inhibitor, 25 mg/kg) or vehicle (DMSO) was intraperitoneally administrated every day for 2 weeks. Similarly, Macitentan (30 mg/kg/day) and Tadalafil (10 mg/kg/day) treatments were started 5 weeks after Sugen injection (once PAH was established) and were administered via gavage alone or in combination with TubA for 2 weeks. Adult knock-out (*Hdac6* ^-/Y^) mice (aged 8 to 10 weeks) and their control groups (C57BL/6 mice) were exposed or not to chronic hypoxia (10% O_2_) for 3 weeks. Mice were genotyped by PCR, as previously described^41^.

### *In vivo* assessment of pulmonary hypertension, RV hypertrophy and pulmonary vascular remodeling

At the time of sacrifice, rats and mice were initially anesthetized with 3–4% isoflurane and maintained with 2% during procedures. Hemodynamic parameters, including RV systolic pressure (RVSP), mean PA pressure (mPAP), cardiac output (CO) and total pulmonary resistance (TPR) were measured blindly by closed chest right heart catheterization (SciSence catheters), as previously described^5, 8, 56^. RV hypertrophy (Fulton index) was measured as the ratio of RV weight to left ventricular plus septal weight (RV/LV + S). Medial wall thickness of distal pulmonary vessels was quantified using Hematoxylin and Eosin (H&E) stain or αSMA labeling, by subtracting the area of the lumen from the total area of the vessel and reported as % of total pulmonary vessel area. Two measurements per vessel in 20 vessels per animal in at least 5 animals per group were performed. The percentage of occluded vessels was calculated by counting 50 vessels per animal, as previously described^57^.

### Statistical analysis

Statistical tests and graphs were done with GraphPad Prism 6.0. Values are expressed as fold change or mean ± standard error of the mean, as they followed a normal distribution. Unpaired Student *t* tests were used for comparisons between 2 groups, and 1-way analysis of variance followed by a Tukey-Kramer post test was used for >2 groups. If the variances of different groups were not equal and depended on the mean of the data, statistical analyses were performed on log transformation of the data. A significance level inferior to 5% (*P* < 0.05) was considered statistically significant.

## Electronic supplementary material

Supplementary data
