# Supplementary material for: HDAC6: A Novel Histone Deacetylase Implicated in Pulmonary Arterial Hypertension
Source: Sci Rep. 2017 Jul 3;7:4546. doi: 10.1038/s41598-017-04874-4 (PMC5495763; doi:10.1038/s41598-017-04874-4)
Supplement: Supplementary file 1 — Supplementary data [file 41598_2017_4874_MOESM1_ESM.pdf]

SUPPLEMENTARY INFORMATION

HDAC6: A Novel Histone Deacetylase Implicated in Pulmonary Arterial Hypertension

Olivier Boucherat, PhD*[1], Sophie Chabot, BSc*[1], Roxane Paulin, PhD[1], Isabelle Trinh, MSc[1], Alice Bourgeois, Bsc[1], François Potus, MSc[1], Marie-Claude Lampron, BSc[1], Caroline Lambert, MSc[1], Sandra Breuils-Bonnet, MSc[1], Valérie Nadeau, PhD[1], Renée Paradis, Bsc[1], Elena A Goncharova, PhD[2], Steeve Provencher, MD, MSc[1], and Sébastien Bonnet, PhD[1]

SUPPLEMENTARY TABLE

	Ctrl (n=13)	PAH (n=15)
Age (years)	43±17	55±15
Gender (female (%))	5 (38.5%)	10 (66.6%)
PAH group		
- HPAH		2 (13.3%)
- IPAH		5 (33.3%)
- Ssc-PAH		7 (46.7%)
- CHD-PAH		1 (6.7%)
Functional class (n (%))		
I	-	0
II	-	1 (6.7%)
III	-	5 (33.3%)
IV	-	7 (46.7%)
Unknown		2 (13.3%)
Pulmonary hemodynamics		
mPAP (mmHg)	-	50±10
CO ($L.min^{-1}$)	-	4.5±0.9
PVR ($dyne.sec^{-1}.cm^{-5}$)	-	722±189
Medication		
Endothelin receptor antagonist	-	8 (53.3%)
PDE5 inhibitor	-	9 (60%)
Prostacyclin analog	-	2 (13.3%)
Unknown	-	4 (26.6%)

Supplementary Table 1. Baseline characteristics of control and PAH patients. Values are means ± SD. HPAH: heritable PAH; IPAH: idiopathic PAH; Ssc-PAH: systemic sclerosis-associated PAH; CHD-PAH: congenital heart disease-associated PAH (Eisenmenger syndrome); mPAP: mean pulmonary arterial pressure; CO: cardiac output; PVR: pulmonary vascular resistance; PDE5: phosphodiesterase-5. Please note than some patients take more than one type of medication.

SUPPLEMENTARY FIGURE LEGENDS

Supplementary Figure 1. HDAC6 is overexpressed in isolated pulmonary artery endothelial cells and right ventricles from PAH patients. A, Western blot and corresponding densitometric analysis of HDAC6 in pulmonary artery endothelial cells (PAEC) isolated from control (n=2) and PAH (n=3) patients. **B,** The expression levels of acetylated-Ku70 (K539) and Ku70 were analyzed by Western blot in PAH-PAECs treated or not with Tubastatin A (1μM), ACY-775 (2μM) or vehicle (DMSO) for 48 hours. **C,** Western blot and corresponding densitometric analysis of HDAC6 in right ventricles (RV) isolated from control (n=3) and PAH (n=5) patients. **E,** Graph showing relative quantification of HDAC6 mRNA expression between control PASMCs (n=3) and PAH-PASMCs (n=3). **E,** Western blot and corresponding densitometric analysis of acetylated-α-Tubulin/α-Tubulin ratio in PASMCs isolated from controls (n=4) and PAH (n=4) patients. Protein expression was normalized by Amido black (AB). ***P<0.001. ns: not statistically significant.

Supplementary Figure 2. HDAC6 is overexpressed in right ventricles from Su/Hx and MCT rats. A, Western blots and corresponding densitometric analyses of HDAC6 and acetylated Ku70 (K539)/Ku70 ratio in right ventricles from controls (n=3), Su/Hx+Veh (n=4) and Su/Hx+TubA (n=5) rats. **B,** Western blots and corresponding densitometric analyses of HDAC6 and acetylated Ku70 (K539)/Ku70 ratio in right ventricles from controls (n=5), MCT+Veh (n=5) and MCT+TubA (n=4) rats. Protein expression was normalized by Amido black (AB). *P<0.05 and **P<0.01.

Supplementary Figure 3. Pharmacological inhibition of HSP90 activity in PAH-PASMCs using 17-AAG mimics AT13387 effects leading to reduced HDAC6 expression. Expression of HDAC6 in PAH-PASMCs was assessed by Western blot after treatment or not with 17-AAG (0.05-1μM) or vehicle (dimethyl sulfoxide) for 48 hours. Blots are representative of two independent experiments.

Supplementary Figure 4. Confirmation of HDAC6 inhibition in PAH-PASMCs. A, The expression levels of HDAC6, acetylated α–Tubulin, α–Tubulin, acetylated Histone H3 and Histone H3 were analyzed by Western blot in PAH-PASMCs treated or not with Tubastatin A (0.15-1μM), ACY-775 (0.25-2μM), siHDAC6 (50nM) or their respective controls for 48 hours. Blots are representative of three independent experiments. **B,** Representative images of control and PAH-PASMCs labeled with Ki67 (proliferation, top panel) and Annexin-V (apoptosis, bottom panel) after treatments with Tubastatin A, ACY-775, siHDAC6 or their respective controls for 48 hours. Scale bar = 50μm. Experiments were performed in triplicate in 3 control and 4 PAH-PASMC cell lines.

Supplementary Figure 5. Inhibition of HDAC6 reduces PAH-PASMCs migration. Graphical view showing the percentage of wound after 24 hours. Experiments were performed in three PAH-PASMC cell lines. ***P<0.001 (*vs* Veh) and #P<0.05 (*vs* siSCRM).

Supplementary Figure 6. Inhibition of HSP90 using siRNA or AT13387 increases acetylation of Ku70 at lysine 539. A, The expression levels of HSP90, acetylated-Ku70 (K539) and Ku70 were assessed in PAH-PASMCs by Western blot after treatments with siHSP90 or siSCRM (50nM for 48 hours). **B,** The expression levels of acetylated-Ku70 (K539) and Ku70 were assessed in PAH-PASMCs by Western blot after treatments with a pharmacological HSP90 inhibitor (AT13387, 10-100nM for 48 hours) or vehicle (DMSO).

Supplementary Figure 7. Inhibition of HDAC6 in serum-starved PAH-PASMCs results in a greater colocalization of Bax with MitoTracker. Representative images of serum-starved PAH-PASMCs treated or not with Tubastatin A (1μM), ACY-775 (2μM) or Veh for 48h and co-stained with MitoTracker Red

and Bax. Colocalization between Bax and MitoTracker was estimated by the Carl Zeiss LSM software using an algorithm that calculates the Pearson's correlation coefficient. Pearson's correlation coefficient was from three independent experiments (three PAH-PASMC cell lines) with ten fields per experiment for a total of 30 fields contributing to the cumulative result. Scale bar=20μm. *P<0.05 (vs NT or Veh).

Supplementary Figure 8. Inhibition of HDAC6 in the Sugen/Hypoxia (Su/Hx) rat model reduces the proportion of occluded pulmonary vessels. The percentage of occluded vessels was calculated by counting 50 vessels per animal (n=5 to 7 rats/group). *P<0.05, ****P<0.0001 (*vs* control) and #P<0.05, and ###P<0.001 (*vs* Su/Hx+Veh).

Supplementary Figure 9. Tubastatin A (TubA) improves hemodynamic parameters, right ventricular hypertrophy and vascular remodeling in the monocrotaline (MCT) rat model. A, Schematic of the experimental design. **B,** Right ventricular systolic pressure (RVSP), mean pulmonary artery pressure (mPAP), cardiac output (CO), total pulmonary vascular resistance (TPR) and right ventricular hypertrophy were measured in control, MCT+Veh (dimethyl sulfoxide) and MCT+TubA (25mg/kg/d) rats; n=7 to 11 rats/group. **C,** Representative images of distal pulmonary vessels stained with Hematoxylin and Eosin (H&E) or labeled with Ki67 (proliferation, red) or TUNEL (apoptosis, red). Vascular smooth muscle cells were labeled using alpha smooth muscle actin (αSMA, green). Graphs represent the degree of vascular remodeling (as determined by the measure of the medial wall thickness and the percent of occluded vessels by H&E stain or αSMA labeling) and the percentage of PASMCs positive for of Ki67 or TUNEL in distal pulmonary vessels. Arrowheads mark positive cells. Scale bar=20μm; n=7 to 11 rats/group. **P<0.01; ***P<0.001; ****P<0.0001 (*vs* control) and #P<0.05, ##P<0.01, and ###P<0.001 (*vs* MCT+Veh).

Supplementary Figure 10. Normoxic or chronically hypoxic *Hdac6* mutant mice exhibit increased acetylation of α-Tubulin and Ku70. The expression levels of acetylated-α-Tubulin, α-Tubulin, acetyl(K539)-Ku70 and Ku70 were measured by Western blot in lungs from wild-type ($Hdac6^{Y/+}$) and *Hdac6* mutant ($Hdac6^{Y/-}$) mice exposed to either normoxia or hypoxia for 3 weeks.

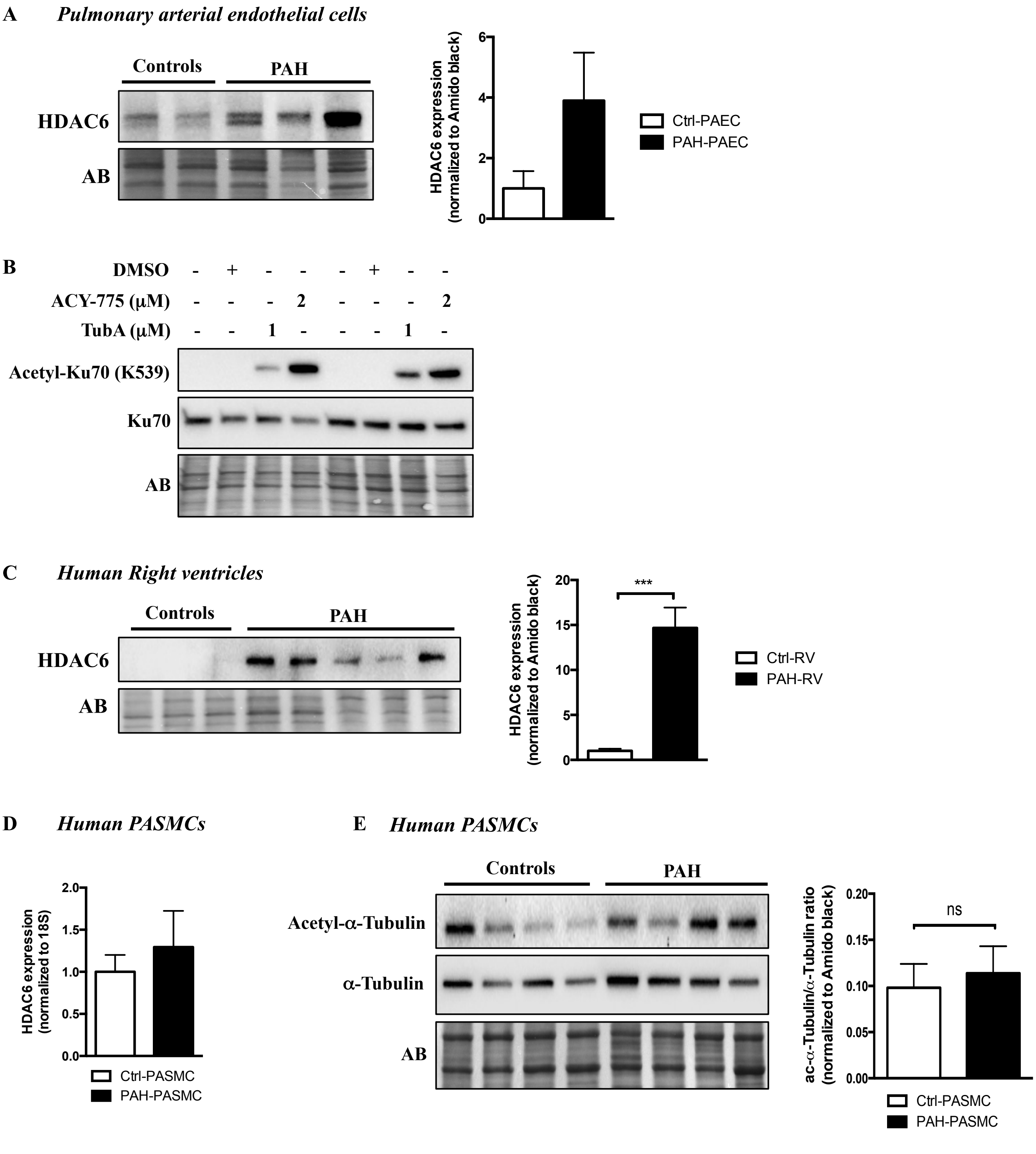

Supplementary Figure 1

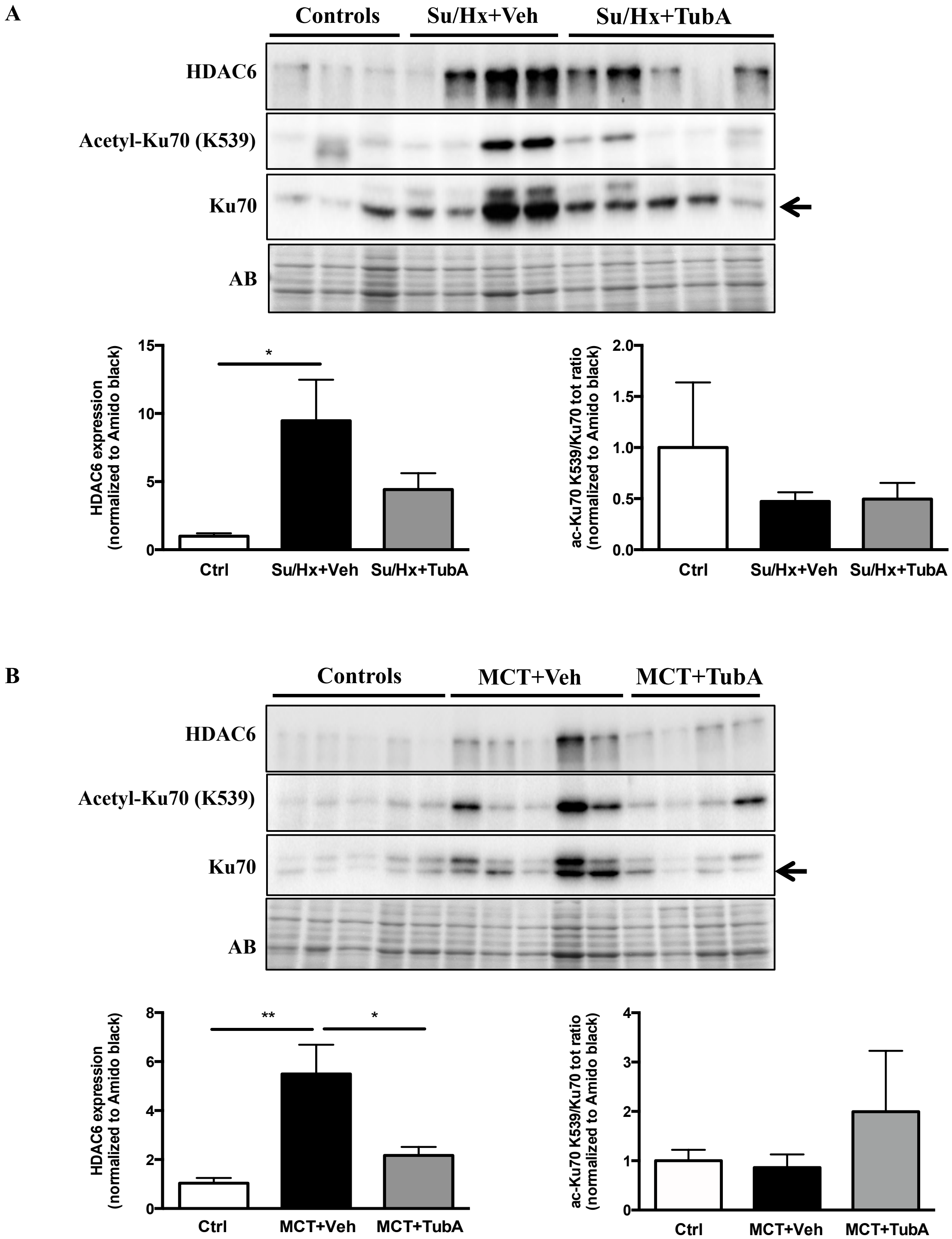

Supplementary Figure 2

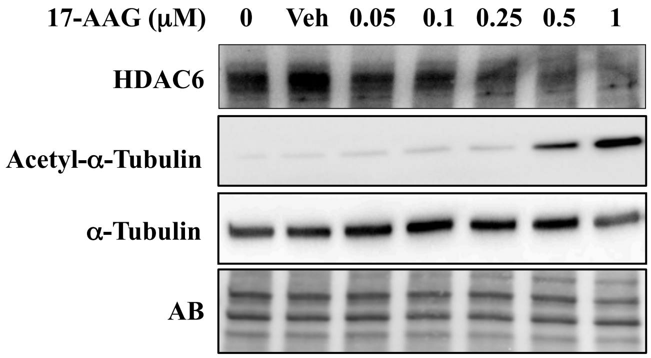

Supplementary Figure 3

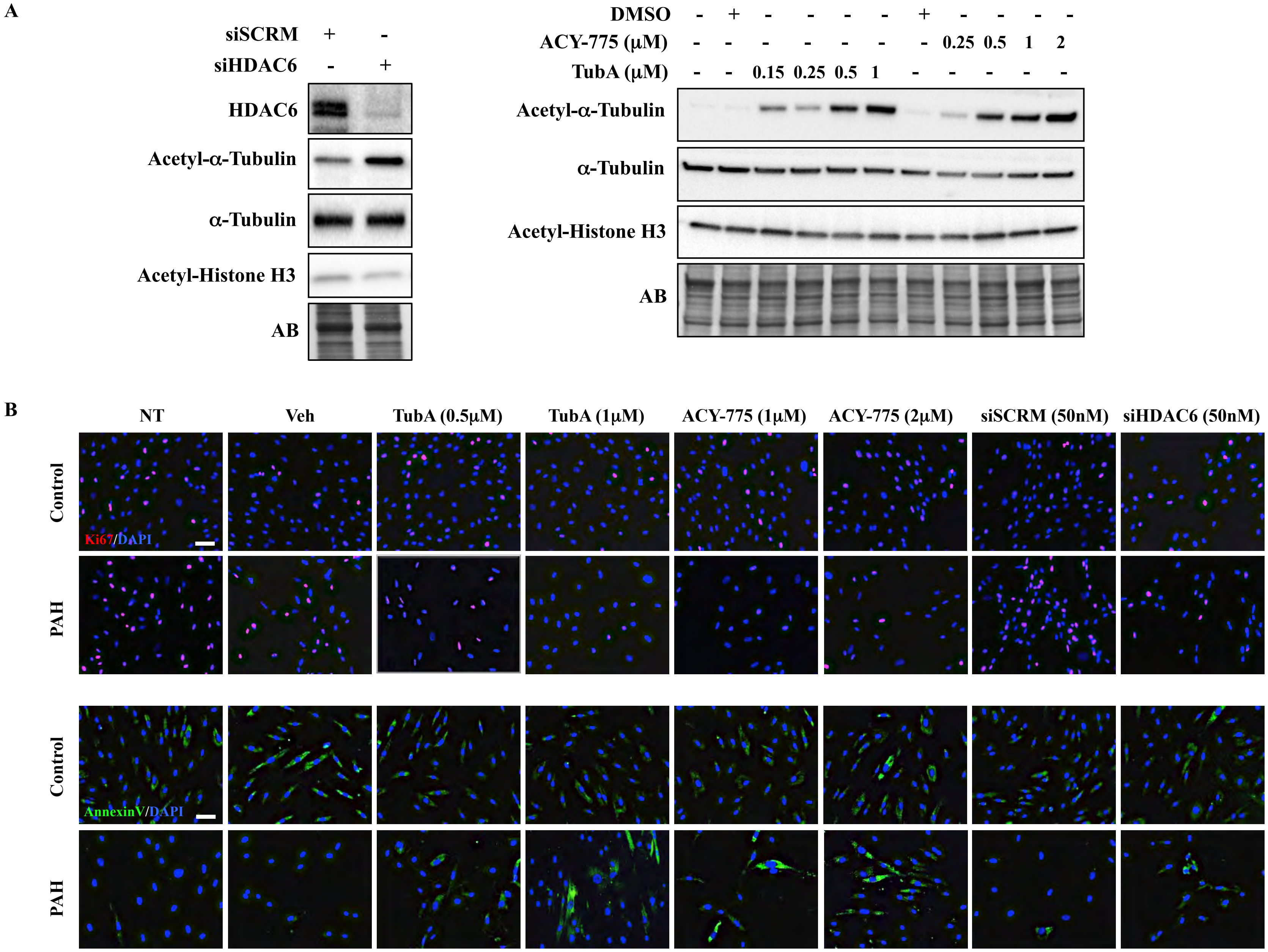

Supplementary Figure 4

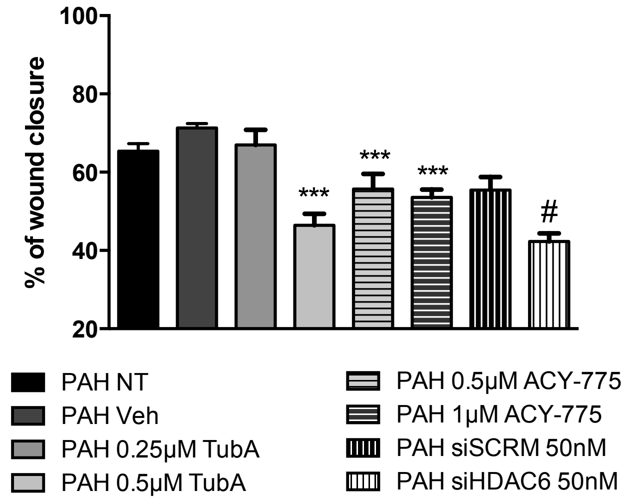

Supplementary Figure 5

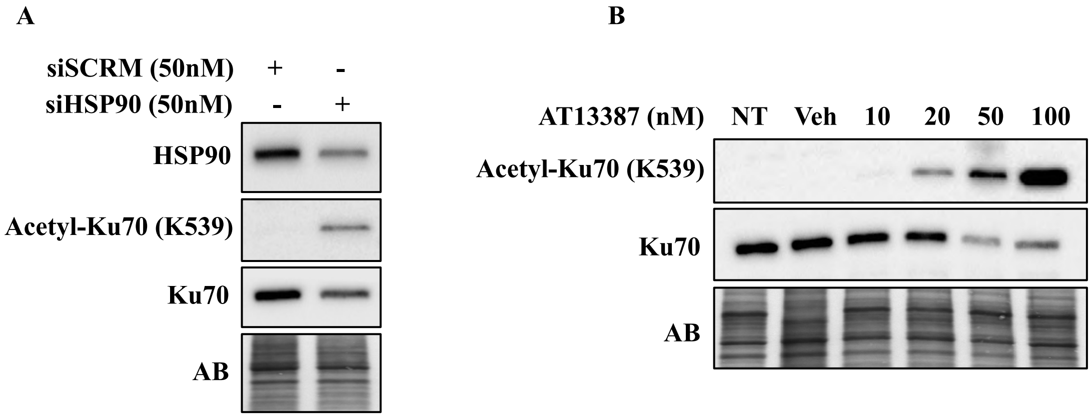

Supplementary Figure 6

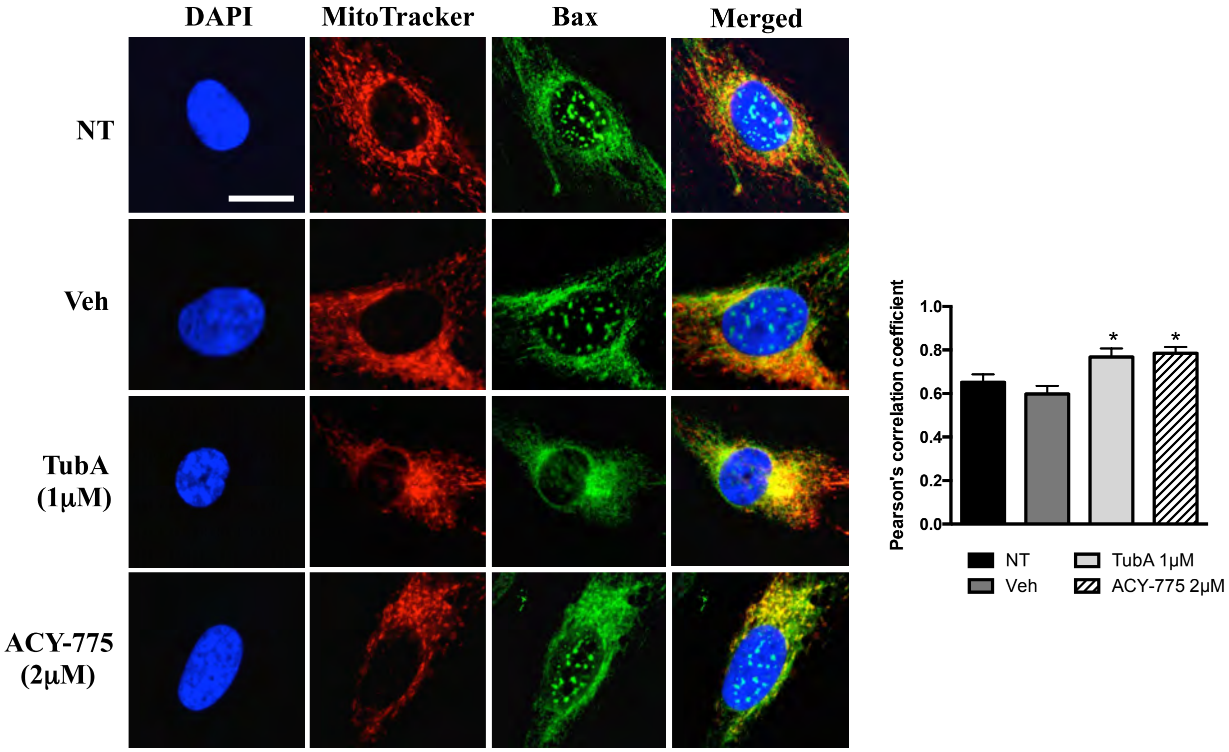

Supplementary Figure 7

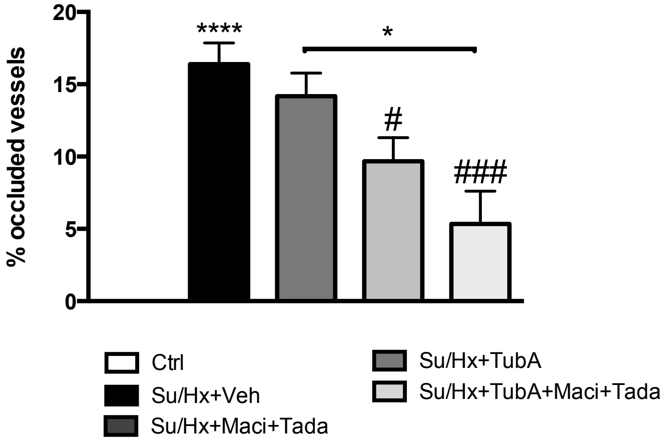

Supplementary Figure 8

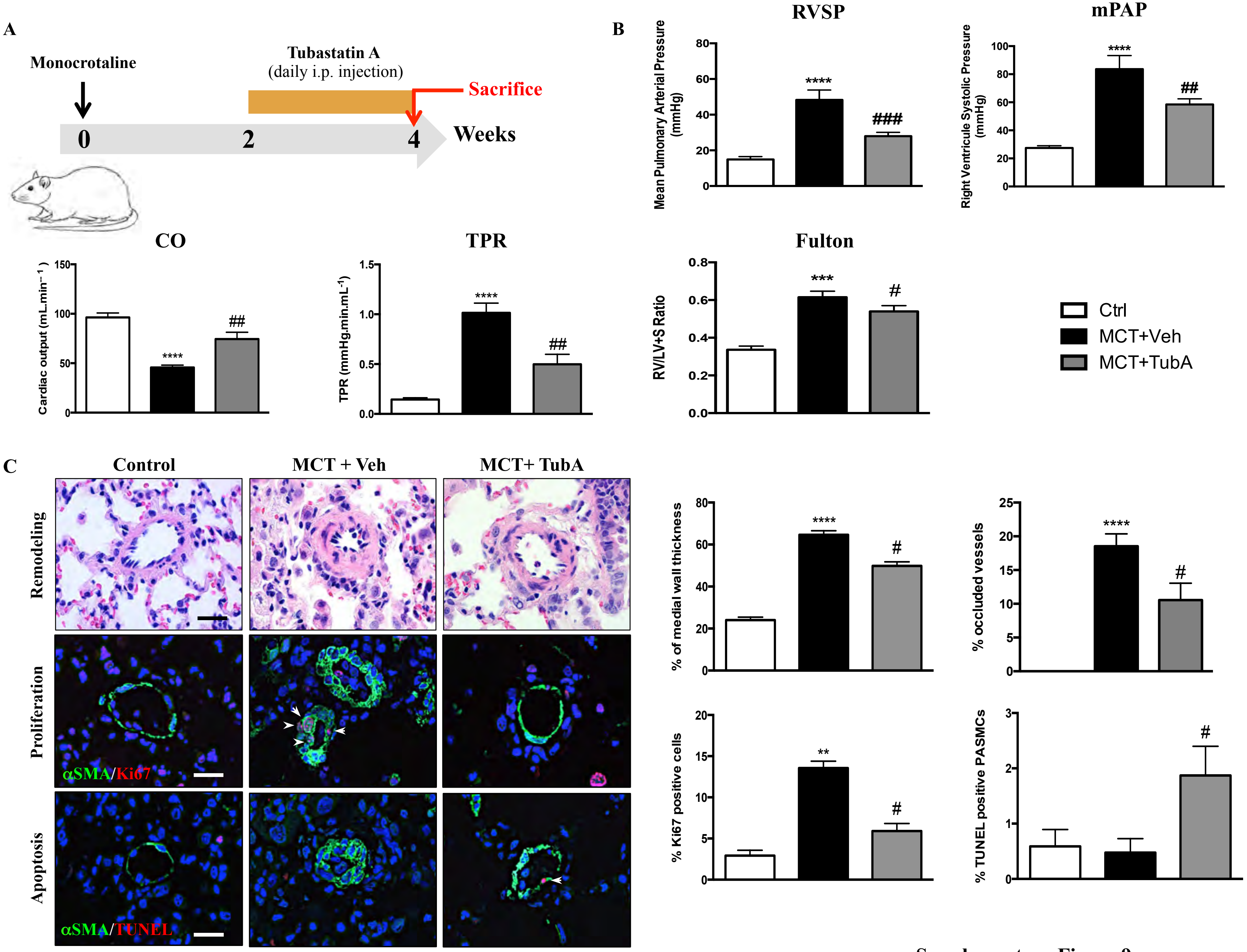

Supplementary Figure 9

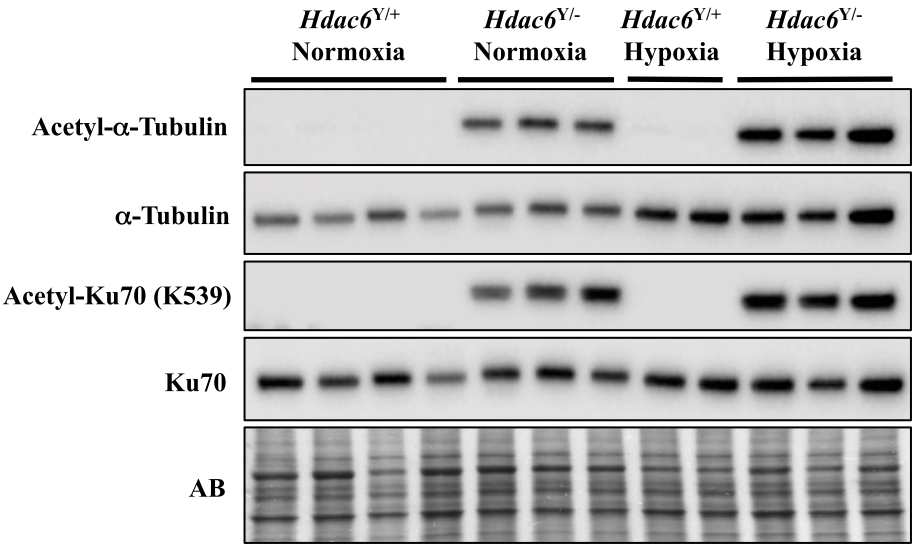

Supplementary Figure 10
